# Patient gender does not influence referral to an orthopaedic surgeon by advanced practice orthopaedic providers: a prospective observational study in Canada

**DOI:** 10.1186/s12913-021-06965-5

**Published:** 2021-09-11

**Authors:** Susan Robarts, Suzanne Denis, Deborah Kennedy, Patricia Dickson, Shahiroz Juma, Veronica Palinkas, Maria Rachevitz, Dragana Boljanovic-Susic, Paul Stratford

**Affiliations:** 1 Holland Orthopaedic and Arthritic Centre, Sunnybrook Health Sciences Centre, 43 Wellesley Street East, Toronto, ON M4Y 1H1 Canada; 2 Department of Physical Therapy, Faculty of Medicine, University of Toronto, Toronto, ON Canada; 3 Department of Occupational Science & Occupational Therapy, Faculty of Medicine, University of Toronto, Toronto, ON Canada; 4 School of Rehabilitation Science, McMaster University, Hamilton, ON Canada

**Keywords:** Advanced practice physiotherapist, Extended scope, Gender bias, Arthroplasty, Physiotherapist, Occupational therapist, orthopaedics, Health equity, Access to care, Gender disparity

## Abstract

**Background:**

The role of an advanced practice physiotherapist has been introduced in many countries to improve access to care for patients with hip and knee arthritis. Traditional models of care have shown a gender bias, with women less often referred and recommended for surgery than men. This study sought to understand if patient gender affects access to care in the clinical encounter with the advanced practice provider. Our objectives were: (1) To determine if a gender difference exists in the clinical decision to offer a consultation with a surgeon; (2) To determine if a gender difference exists in patients’ decisions to accept a consultation with a surgeon among those patients to whom it is offered; and, (3) To describe patients’ reasons for not accepting a consultation with a surgeon.

**Methods:**

This was a prospective study of 815 patients presenting to a tertiary care centre for assessment of hip and knee arthritis, with referral onward to an orthopaedic surgeon when indicated. We performed a multiple logistic regression analysis adjusting for severity to address the first objective and a simple logistic regression analysis to answer the second objective. Reasons for not accepting a surgical consultation were obtained by questionnaire.

**Results:**

Eight hundred and fifteen patients (511 women, 304 men) fulfilled study eligibility criteria. There was no difference in the probability of being referred to a surgeon for men and women (difference adjusted for severity = − 0.02, 95% CI: − 0.07, 0.02). Neither was there a difference in the acceptance of a referral for men and women (difference = − 0.05, 95% CI: − 0.09, 0.00). Of the 14 reasons for declining a surgical consultation, 5 showed a difference with more women than men indicating a preference for non-surgical treatment along with fears/concerns about surgery.

**Conclusions:**

There is no strong evidence to suggest there is a difference in proportion of males and females proceeding to surgical consultation in the model of care that utilizes advanced practice orthopaedic providers in triage. This study adds to the evidence that supports the use of suitably trained alternate providers in roles that reduce wait times to care and add value in contexts where health human resources are limited. The care model is a viable strategy to assist in managing the growing backlog in orthopaedic care, recently exacerbated by the COVID-19 pandemic.

**Supplementary Information:**

The online version contains supplementary material available at 10.1186/s12913-021-06965-5.

## Background

Osteoarthritis (OA) of the hip and knee is a disabling condition when in advanced stages [1, 2]. Total hip arthroplasty and total knee arthroplasty are successful surgical procedures undertaken to relieve pain and restore function. In Canada, more than 137,000 patients undergo such procedures annually [3], and yet, patients with advanced arthritis continue to experience significant delays in accessing care, specifically a consultation with an orthopaedic surgeon. A report published by the Fraser Institute shows orthopaedics as having some of the longest waits among 12 medical specialties in Canada [4]. Patients now face even longer waits due to the COVID-19 pandemic which saw hospitals directed to substantially reduce elective, but medically necessary, surgeries to preserve health care resources, resulting in a growing backlog in orthopaedic care globally [5–8].

Over the past decade, models of care utilizing advanced practice physiotherapists (APPs) to assess and prioritize patients have been adopted and spread to improve access and care for orthopaedic patients [9–11]. Research has shown that suitably trained physiotherapists in an advanced practice role improve access to care and add value to the clinic visit by identifying patients that require a consultation with a surgeon, and diverting those that do not, to alternate treatment options, and by providing patients with personalized education and advice [12–17]. Level of agreement between APPs and orthopaedic surgeons on diagnosis and care decisions has been found to be high, and patients are highly satisfied with the technical skills, personal manner, information received and APP visit overall [18–22]. While a large body of research has accumulated regarding the clinical effectiveness of the APP role in the UK, Canada, Australia, and other countries, there is currently no information on unintended negative consequences of the model of care which are necessary to explore for its sustainability.

The APP’s role in evaluating which patients require surgical consultation and which patients do not is a complex process that involves a detailed examination of patient characteristics and a discussion with the patient regarding their knowledge of OA and views on joint replacement [16, 23, 24]. Previous research exploring the rate of use of total joint replacement found that women were less likely to be recommended a surgical approach to care by their primary care physicians as well as orthopaedic surgeons [25–27]. Further, Borkhoff et al. demonstrated that gender bias influences elements of informed decision-making during the clinical encounter, with poorer physician performance when the patient was a woman, and less time given to women in the encounter [26]. These findings are concerning in that the APP is an additional point of assessment for patients with moderately advanced arthritis and the potential for gender bias exists. An important goal for the clinic visit with the advanced practice provider is that patients with similar clinical characteristics receive the same information and treatment options regardless of gender.

Since inception of our model of care, we have employed an evaluation strategy that uses both quality improvement methodology and formal research. As part of our quality approach, we conducted a retrospective review (unpublished) on patients presenting to the clinic between 2011 and 2014, where women appeared less likely to proceed to a consultation with a surgeon. This may have occurred for 1 of 2 reasons: either women were less likely to be recommended a consultation with a surgeon or, women were less likely to accept the referral. Due to gaps in clinical documentation, we were unable to identify if this reflected a gender disparity in utilization of specialty care; furthermore, that analysis did not take disease severity into account.

The advanced practice provider’s assessment integrates complex information across several aspects including clinical findings, functional status, and results of diagnostic imaging. It is important to understand what factors within these aspects predict the offer of a consultation with a surgeon and to examine gender as one of the factors that may influence treatment recommendations. Possible explanations for the gender gap include patient perceptions about surgery (and associated risks, benefits, and recovery), personal preferences, availability of social support, caregiving responsibilities, and the patient-provider interaction [24, 28, 29].

### Study objectives

Our study had three objectives: (1) To determine if a gender difference exists in the clinical decision to offer a consultation with a surgeon; (2) To determine if a gender difference exists in patients’ decisions to accept a consultation with a surgeon among those patients to whom it is offered; and, (3) To describe patients’ reasons for not accepting a consultation with a surgeon. The conclusions will inform the development of mitigating strategies and be useful to decision-makers in Canada and other countries with Universal coverage and where access to care is a significant issue necessitating development of alternate models of care.

## Methods

### Study design

This prospective study analysed data from 815 consecutive patients fulfilling this study’s eligibility criteria and presenting to a tertiary care centre in Ontario, Canada for initial assessment of moderate to advanced hip and knee arthritis. Participation was voluntary and all participants provided informed consent. Approval for use of human subjects was obtained from the Research Ethics Board of Sunnybrook Health Sciences Centre.

### Setting and participants

Patients are referred to the tertiary care centre by primary care physicians, nurse practitioners, and physician specialists for consideration of arthroplasty and undergo a comprehensive assessment with an advanced practice provider (Physiotherapist or Occupational Therapist) in the outpatient clinic within 4 weeks from referral. The advanced practice provider evaluates the patient’s condition and facilitates referral onward to an orthopaedic surgeon when indicated. Patients were eligible to participate in the study if they were attending the clinic for initial assessment and, the reason for referral indicated moderate to advanced hip and knee arthritis. Patients were excluded from the study if they were returning for a follow up visit, unable to independently complete questionnaires, and if they presented for second opinion on prior total hip or knee replacement which is an additional function of the clinic.

During the initial assessment, the advanced practice provider performs a history and physical examination and incorporates results from standardized patient reported and performance outcome measurement, and diagnostic tests. The providers discuss the results of the assessment and treatment options with the patient, and together with the patient, devise a management plan, including referral to an orthopaedic surgeon.

### Advanced practice providers

These healthcare professionals are typically physiotherapists with advanced formal education (beyond entry to practice) and extensive orthopaedic experience. We have one occupational therapist among 6 physiotherapists in the role. The advanced practice providers function independently upon successful completion of a 3-month Practice Development Program which is a workplace-based structured learning process, with competency assessment utilizing tools developed at our centre and those developed by colleagues in Australia [13, 30, 31]. Advanced practice providers have an extended scope of practice and are equipped to order X-rays, laboratory tests, and other investigations through Medical Directives.

### Standardized clinical prioritization

The advanced practice providers use a tool developed by our centre for prioritizing patients (the Severity Scoring System) which acts as a guide to interpreting findings across three main elements of the standardized assessment: clinical findings, functional findings, and radiological findings. The clinical and functional findings are scored across 4 severity levels (e.g. no findings, mild, moderate, and severe) and scored from a rating of 0 to 3. Clinical findings include a patient-reported standardized outcome measure for pain intensity, the P4 [32]. Functional findings include results from the following standardized outcome measures recommended in the assessment of patients with hip and knee OA: patient-reported function with the Lower Extremity Functional Scale (LEFS); 30s Chair Stand Test; 40 m Fast Paced Walk Test; and, Timed Up and Go in lower functioning patients [33]. Radiological findings are scored across 3 severity levels (e.g. no findings, mild/moderate, and marked/advanced) and scored from a rating of 0 to 2 given the known lack of association between radiological findings and pain and disability [34]. The data coding for radiological findings had 4 categories (Kellgren-Lawrence grade) [35]. The Total Severity Score out of 8 is then aligned with urgency and priority levels to assist the clinician in determining an appropriate management plan.

### Reasons for declining surgical consultation and surgery

The project team developed a simple questionnaire for participants declining the offer of surgical consultation to indicate their reasons for not proceeding, so as to explore patient preferences as a potential factor in gender disparity (see Additional file 1). The list of reasons was informed by the literature and confirmed with patients through an initial trial period until saturation in items was reached [24, 29, 36].

### Data analysis

We summarized data as frequency counts and percentages, means and standard deviations or quartiles depending on the data’s parametric properties. For the first objective that addresses a potential gender difference in the patients referred forward, we performed three logistic regression analyses with the dependent variable being the decision to refer to surgeon (yes, no). Gender was the only independent variable in the first analysis. The second analysis examined the effect of gender having adjusted for joint type, age, radiological findings, P4, LEFS, 30s Chair Stand Test, and trial of conservative treatment. The third analysis examined the effect of gender having adjusted for the total severity score. We applied a simple logistic regression analysis for the second objective that examined a gender difference in the proportion of patients accepting a referral with the orthopaedic surgeon. We estimated gender specific probabilities of referral (1st objective) and acceptance (2nd objective), and their 95% confidence intervals. We applied a critical *p*-value of 0.05 for hypotheses tests. Data were analyzed using STATA v16.1 (StataCorp, College Station, Texas).

**Objective 1:** Sample size estimates for multiple logistic regression analyses are typically estimated by considering the expected number of events per variable (EPV) [37]. Our sample size estimate was based on 8 independent variables (7 covariates plus gender), 20 events per variable, and 20% of patients not being referred forward. These assumptions produced a sample size of 800 patients. **Objective 2:** We anticipated that 85% of patients offered a consultation with the surgeon would accept. The assumptions for the sample size calculation were: Type 1 error 0.05 2-tailed; Type II error 0.20; anticipated proportion of patients accepting referral in the larger proportion group = 0.90; anticipated difference in proportion 0.10 (i.e., smaller proportion = 0.80). These assumptions yielded a sample size of 398 patients.

## Results

Eight hundred fifteen patients (511 women, 304 men) fulfilled our study’s eligibility criteria. Table 1 provides a summary of the participants’ characteristics and sample sizes when data were available for fewer than the entire sample. Women and men were of similar age and BMI. Women and men also had similar clinical, functional, and radiological severity scores. As would be expected, men had slightly faster times for the performance measures.

**Table 1:** Descriptive Statistics

Characteristic or Measure	Women *N* = 511	Men *N* = 304
Age mean (sd)	66.4 (9.9)	66.5 (10.2)
BMI mean (sd)	30.4 (8.2)	30.2 (5.7)
Joint hip / knee	201 / 310	122 / 182
(% hip / % knee)	(39.3% / 60.7%)	(40.1% / 59.9%)
Comorbidities quartiles; n	1, 2, 3; 511	1, 2, 3; 303
Failed Conservative Treatment n/N (%)	212/492 (43.1%)	138/295 (46.8%)
Kellgren-Lawrence Score		
1 n (%)	3 (0.6)	1 (0.3)
2 n (%)	121 (23.7)	53 (17.4)
3 n (%)	95 (18.7)	41 (13.5)
4 n (%)	292 (57.1)	209 (68.8)
Clinical score (0–3) quartiles; n	1.5, 2.0, 2.0; 510	2.0, 2.0, 2.0; 304
Functional score (0–3) quartiles; n	1.5, 2.0, 2.0; 510	1.0, 2.0, 2.0; 304
Radiological score (0–2) quartiles; n	1.5, 2.0, 2.0; 510	1.5, 2.0, 2.0; 304
Total severity score (0–8) quartiles; n	4.5, 5.5, 6.0; 510	5.0, 5.5, 6.0; 304
P4 pain score (0–40) mean, sd; n	23.9, 9.8; 509	22.4, 9.7; 303
LEFS score (0–80) mean, sd; n	31.9, 16.2; 509	32.1, 16.1; 303
30s Chair Stand mean, sd; n	10.3, 4.3; 425	11.2, 4.7; 267
40 m walk time mean, sd; n	35.1, 10.8; 436	32.8, 12.6; 272
Timed-up-and-go quartiles; n	20, 25, 40; 58	16, 24, 30; 22

Three analyses—one unadjusted and two adjusted—were performed to estimate the probabilities of women and men being referred to a surgeon (Table 2). All three analyses yielded between gender differences that were neither statistically significant (*p* > 0.05) nor met our a priori specification of an important difference (i.e., a difference in proportions > 0.10). For the unadjusted analysis the proportion of women offered a surgical referral was 0.75 (382/511) compared to 0.79 (241/304) for men (difference = − 0.04, 95% CI: − 0.10, 0.01). Six hundred sixty-two patients (404 women, 258 men) had complete data for the analysis that adjusted for joint, age, Kellgren-Lawrence score, P4 score, LEFS score, 30s chair stand score, trial of conservative treatment. The adjusted probability of being referred to a surgeon was virtually identical for women and men (difference = − 0.01, 95% CI: − 0.06, 0.04). For the analysis that adjusted for Total Severity Score, complete data were available for 814 patients (510 women, 304 men). Once again, the adjusted probabilities of being referred to a surgeon were nearly identical for women and men (difference = − 0.02, 95% CI: − 0.07, 0.02).

**Table 2:** Probability of Being Referred to Surgeon

	Probability of Being Referred to Surgeon		
	Unadjusted *n* = 815	Adjusted for 7 Covariates^a^ *n* = 662	Adjusted for Total Severity Score *n* = 814
Women (95% CI)	0.75 (0.71, 0.78)	0.72 (0.69, 0.75)	0.76 (0.73, 0.79)
Men (95% CI)	0.79 (0.75, 0.84)	0.73 (0.69, 0.77)	0.78 (0.74, 0.81
Difference (95% CI)	−0.04 (− 0.10, 0.01)	− 0.01 (− 0.06, 0.04)	− 0.02 (− 0.07, 0.02)
Test statistic, p-value	Z = 1.50, *p* = 0.13	Z = 0.30, *p* = 0.76	Z = 0.95, *p* = 0.34

^a^Covariates were: joint, age, Kellgren-Lawrence score, P4 score, LEFS score, 30s chair stand score, trial of conservative treatment

Our second objective was to determine if there was a difference in the proportion of women and men accepting a referral to a surgeon. Of the 815 patients assessed, 609 were offered a referral (Table 3). The proportion of women accepting a surgical referral was 0.88 (329/373) compared to 0.93 (219/236) for men. This difference (difference = − 0.05, 95% CI: − 0.09, 0.00) was neither statistically significant (*p* > 0.05) nor did it meet our standard for an important between gender difference of |0.10|.

**Table 3:** Probability of Accepting Referral to Surgeon

	Probability of Accepting Referral to Surgeon *n* = 609
Women (95% CI)	0.88 (0.85, 0.91)
Men (95% CI)	0.93 (0.89, 0.96)
Difference (95% CI)	−0.05 (− 0.09, 0.00) Z = 1.84, *p* = 0.0.07

Table 4 summarizes the reasons for not accepting a surgical referral. Five items where the endorsement proportions differed by 0.10 or more and their respective confidence intervals excluded zero were: prefer no surgery, prefer non-surgical treatments, afraid of surgery, surgery may not help or may make worse; and, burden on others.

**Table 4:** Reasons for Declining Referral

Reason	Proportion Endorsing Response F: M	Difference in Proportions (95% CI)
Prefer no surgery	0.41: 0.16	**−0.25 (− 0.48, − 0.03)**
Need more time to consider	0.36: 0.47	0.11 (− 0.16, 0.39)
Pain not bad enough	0.39, 0.42	0.03 (− 0.24, 0.30)
Able to do activities that matter to me	0.35, 0.32	− 0.03 (− 0.29, 0.22)
Not ready, surgery is a last resort	0.61, 0.37	− 0.24 (− 0.51, 0.02)
Prefer non-surgical treatments	0.41, 0.10	**− 0.30 (− 0.51, − 0.10)**
Afraid of surgery	0.22, 0.05	**−0.17 (− 0.33, − 0.01)**
Health not good enough	0.02, 0.05	0.03 (− 0.08, 0.13)
Current treatments helping	0.15, 0.10	−0.05 (− 0.23, 0.12)
Surgery may not help or may make worse	0.10, 0	**−0.10 (− 0.20, − 0.01)**
Lack support for my recovery	0.02, 0.10	0.08 (− 0.07, 0.23)
Burden on others	0.13, 0	**−0.13 (− 0.23, − 0.02)**
Caregiver or other family responsibilities	0.08, 0	−0.08 (− 0.16, 0.01)
Can’t take time off work for surgery	0.03, 0.05	0.02 (−0.08, 0.13)

## Discussion

Our study has shown that there was no difference in the probability of being referred to a surgeon for men and women with hip and knee arthritis by advanced practice orthopaedic providers in a tertiary care setting. This finding adds support for the model of care as a pathway to optimize access to care and treatment. At the core of this research is an inquiry into whether there is gender disparity in patients referred to surgeons by advanced practice providers as potentially indicated in our previous research. Gender disparity, due to gender bias influencing clinical decision-making, is well documented in traditional routes of care for patients with hip and knee arthritis, with women less often referred for surgical consultation and surgical treatment [25, 27, 38, 39]. In addition, the patient-physician interaction is known to be suboptimal for women with respect to informed decision-making and physician interpersonal behaviour [26]. In our model of care utilizing appropriately qualified and trained alternate advanced practice providers, women were not disadvantaged in receiving care.

It is interesting to note that 5 of the 14 reasons for declining a consultation with a surgeon show a gender difference: more women than men indicated a preference for non-surgical treatment along with fears/concerns about surgery. Additionally, women indicated they did not want to be a burden on others. These findings substantiate what has been documented in prior research; women wait longer for surgery, are fearful, and are concerned for their caregiving roles [29, 40]. These are important points of discussion when presenting treatment options to women. Women hesitant regarding surgery may require additional medical information (risks and benefits of surgery), information regarding the recovery trajectory and how much help/what sort of help would be needed following surgery, in addition to when usual activities can be resumed. These topics are key elements of informed decision making which was shown to be lacking in the traditional model of care, particularly in clinical encounters with women [26, 41]. While informed decision making was not specifically examined in the current study, the advanced practice providers are trained to encourage participation in the discussion regarding treatment options and explore patient preferences, and have additional clinical time to do so; furthermore, these providers are trained to gauge patient understanding. These enhancements to the traditional model of care likely improve the quality of the assessment and contribute to our study findings.

Equitable access to care through standardized assessment is the rationale for implementation of this model of care in Ontario, Canada [13, 42]. The model of care includes supporting elements that are integral to its performance such as standardized role entry qualifications, standardized training with competency assessment, standardized clinical assessments, and the incorporation of outcome measurement – both patient reported and performance measures, in addition to an emphasis on shared decision-making regarding treatment options that include self-management and non-surgical best practice recommendations. The model of care, as designed, does not appear to negatively impact women in accessing a surgical consultation or surgical treatment, and continues to show promise as a sustainable strategy that improves access to quality care and adds value when utilizing experienced healthcare professionals with a complementary skillset in triage.

Wait times have been made worse by the COVID-19 pandemic and a backlog of elective surgeries is now experienced in many countries that were forced to pause non urgent services to maintain health system capacity. The model of care is a viable strategy to address the backlog in orthopaedic care through effective prioritization of patients, and is consistent with service recovery recommendations [43]. In countries with universal health care systems, alternate models of care such as this are required to assist with resumption of services and improving access to care.

### Limitations

This study is the first to look at gender bias in a model of care utilizing alternate orthopaedic providers, and as such makes an important contribution to the body of literature on models of care in orthopaedics and arthritis. A potential limitation of the study is that it was conducted in a single academic hospital (the developers of the model of care) in Ontario; however, results can be generalized to other sites that have applied and maintained the key characteristics of the model of care. This team has had particular interest in gender bias and shared decision-making. If bias were found in these advanced practice providers, then it’s likely that similar or greater bias would be found in advanced practice providers elsewhere and would warrant further exploration and mitigating strategies. The advanced practice providers were aware of the study objectives; which, in addition to the team’s special interest in gender bias, likely contributed to the positive outcome. Further research examining shared decision-making would be beneficial to examine with advanced practice providers.

## Conclusion

Gender does not appear to play a role in access to care when patients are assessed by experienced, suitably-trained advanced practice providers suggesting an improvement in the quality of the assessment and patient-clinician interaction from the traditional model of care.

## Supplementary Information

**Additional file 1.** Patient Questionnaire: Reasons for not proceeding to see a surgeon.

## Data Availability

The dataset generated and analysed during the current study are available from the corresponding author on reasonable request.

## Abbreviations

APP: Advanced practice physiotherapist
LEFS: Lower Extremity Functional Scale
BMI: Body Mass Index
