# Supplementary material for: Patient gender does not influence referral to an orthopaedic surgeon by advanced practice orthopaedic providers: a prospective observational study in Canada
Source: BMC Health Serv Res. 2021 Sep 11;21:952. doi: 10.1186/s12913-021-06965-5 (PMC8435171; doi:10.1186/s12913-021-06965-5)
Supplement: Supplementary file 1 — Additional file 1. Patient Questionnaire: Reasons for not proceeding to see a surgeon. [file 12913_2021_6965_MOESM1_ESM.docx]

**Additional file 1**

**Patient Questionnaire: Reasons for not proceeding to see a surgeon**

| Since you are not going on to see a surgeon at this time, please select the reasons from the list below.  **Circle the number that applies. You may circle more than one number:** |
| --- |
| 1. I prefer not to have surgery |
| 1. I need more time to consider |
| 1. The pain is not bad enough |
| 1. I am able to do the activities that matter to me |
| 1. I am not ready yet, surgery is a last resort |
| 1. I prefer non-surgical treatments |
| 1. I am afraid of surgery |
| 1. My health is not good enough |
| 1. Current treatments are helping |
| 1. Surgery may not help me or may make me worse |
| 1. I lack support for my recovery |
| 1. I don’t want to be a burden on others |
| 1. I am a caregiver, or have other family responsibilities |
| 1. I can’t take time off work for surgery |
| 1. I have concerns related to COVID-19 |
| 1. Other: |
